# Evaluation of X-ray and carbon-ion beam irradiation with chemotherapy for the treatment of cervical adenocarcinoma cells in 2D and 3D cultures

**DOI:** 10.1186/s12935-022-02810-9

**Published:** 2022-12-09

**Authors:** Kazumasa Sekihara, Hidetomo Himuro, Nao Saito, Yukihide Ota, Taku Kouro, Yohsuke Kusano, Shinichi Minohara, Ryoichi Hirayama, Hiroyuki Katoh, Tetsuro Sasada, Daisuke Hoshino

**Affiliations:** 1 Department of Cancer Biology, Kanagawa Cancer Center Research Institute, 2-3-2, Nakao, Asahi-ku, Yokohama, Kanagawa 241-8515 Japan; 2 Biospecimen Center, Kanagawa Cancer Center, Yokohama, 2418515 Japan; 3 Department of Cancer Immunotherapy, Kanagawa Cancer Center Research Institute, Yokohama, 2418515 Japan; 4 Department of Radiation Oncology, Kanagawa Cancer Center, Yokohama, 2418515 Japan; 5 Department of Obstetrics and Gynecology, Yokohama City University Graduate School of Medicine, Yokohama, 2360004 Japan; 6 Section of Medical Physics and Engineering, Kanagawa Cancer Center, Yokohama, 2418515 Japan; 7 Department of Charged Particle Therapy Research, Institute for Quantum Medical Science (iQMS), National Institutes for Quantum Science and Technology (QST), Chiba, 2638555 Japan

**Keywords:** 3D spheroid, Carbon-ion beam, Hypoxia, cancer stem cell, Cervical adenocarcinoma

## Abstract

**Background:**

Cervical cancer is the second most common cancer in women and causes more than 250,000 deaths worldwide. Among these, the incidence of cervical adenocarcinomas is increasing. Cervical adenocarcinoma is not only difficult to detect and prevent in the early stages with screening, but it is also resistant to chemotherapy and radiotherapy, and its prognosis worsens significantly as the disease progresses. Furthermore, when recurrence or metastasis is observed, treatment options are limited and there is no curative treatment. Recently, heavy-particle radiotherapy has attracted attention owing to its high tumor control and minimal damage to normal tissues. In addition, heavy particle irradiation is effective for cancer stem cells and hypoxic regions, which are difficult to treat.

**Methods:**

In this study, we cultured cervical adenocarcinoma cell lines (HeLa and HCA-1) in two-dimensional (2D) or three-dimensional (3D) spheroid cultures and evaluated the effects of X-ray and carbon-ion (C-ion) beams.

**Results:**

X-ray irradiation decreased the cell viability in a dose-dependent manner in 2D cultures, whereas this effect was attenuated in 3D spheroid cultures. In contrast, C-ion irradiation demonstrated the same antitumor effect in 3D spheroid cultures as in 2D cultures. In 3D spheroid cultures, X-rays and anticancer drugs are attenuated because of hypoxia inside the spheroids. However, the impact of the C-ion beam was almost the same as that of the 2D culture, because heavy-particle irradiation was not affected by hypoxia.

**Conclusion:**

These results suggest that heavy-particle radiotherapy may be a new therapeutic strategy for overcoming the resistance of cervical adenocarcinoma to treatment.

**Supplementary Information:**

The online version contains supplementary material available at 10.1186/s12935-022-02810-9.

## Introduction

Cervical cancer is the second most common cancer in women worldwide, with 500,000 new cases annually, resulting in more than 250,000 deaths [1]. Most cervical cancers are HPV-related, including squamous cell carcinoma (70%) and adenocarcinoma (25%) [2]. The incidence of adenocarcinoma has markedly increased, especially among younger women, although the incidence of squamous cell carcinoma has declined [3, 4]. It has been shown that adenocarcinoma has a worse prognosis and lower survival rate than squamous cell carcinoma because of the high rate of metastases and resistance to chemoradiotherapy [3]. Therefore, new therapeutic strategies are required to improve the survival of patients with cervical adenocarcinoma.

Recently, heavy-particle radiotherapy using carbon ions (C-ions) has attracted a great deal of attention because of its higher probability of tumor control and ability to minimize damage to surrounding normal cells compared to conventional radiotherapy using X-rays [5]. C-ion radiotherapy has also been used to treat cervical adenocarcinoma. Relatively good results have been reported compared to cisplatin (CDDP)-based concurrent chemoradiotherapy, which is established as a standard treatment, and image-guided brachytherapy [6, 7]. In addition, clinical trials are ongoing to combine C-ion radiotherapy with CDDP or image-guided brachytherapy for locally advanced cervical cancer [8, 9]. Carbon beam irradiation has been reported to kill cancer stem cells (CSCs) better than X-ray irradiation in colon cancer [10] and inhibits invasion and metastasis in mouse osteosarcoma [11]. Thus, C-ion radiotherapy has the potential to overcome the therapeutic resistance of cervical adenocarcinoma; however, our knowledge regarding cervical adenocarcinoma is still limited.

For almost half a century, two-dimensional (2D) cell cultures have been used to evaluate the efficacy of radiation and drugs. However, 2D monolayer culture systems cannot closely reflect the situation of tumors in vivo because of the loss of proper tumor architecture and cell-cell contact. However, using three-dimensional (3D) cell cultures has become more popular in recent years because they can mimic the tumor environment better than a 2D monolayer cultures [12, 13]. Among several systems for 3D cell culture [14], spheroids are the most straightforward and widely used because they are easy to cultivate, have been shown to reproduce the effects of radiotherapy and chemotherapy, and can mimic tumors in vivo, such as tissue structure and gradients of oxygen and nutrients [15].

Taken together, accurately evaluating the efficacy of heavy-particle radiotherapy for cervical adenocarcinoma requires the use of a 3D model that better reproduces the in vivo tumor environment. However, no such report has been published at present. To our knowledge, this study is the first to demonstrate that C-ion beam kills CSCs and hypoxic areas in cervical adenocarcinoma. We also demonstrated that the spheroid culture systems are suitable as an evaluation system in the field of radiotherapy.

## Materials and methods

### Cell cultures and reagents

Two representative cervical adenocarcinoma cell lines, HeLa and HCA-1 were obtained from the Japanese Collection of Research Bioresources (JCRB). A cervical squamous cell carcinoma cell line, SiHa was kindly provided by Prof. Mitomu Kioi, Yokohama City University Graduate School of Medicine (Yokohama, Japan). HeLa (HPV-18 positive) and SiHa (HPV-16 positive) cells contain wild-type p53, while HCA-1 cells express mutated p53 (R273C). Cells were cultured in Dulbecco’s Modified Eagle’s Medium (DMEM) (Fujifilm Wako Chemicals, Osaka, Japan) supplemented with 10% fetal bovine serum (FBS) (Hyclone, Cytiva, Marlborough, MA, USA), 100 U/mL of penicillin, and 100 mg/mL of streptomycin and then incubated at 37 ℃ in a humidified 5% CO_2_ atmosphere. CDDP and paclitaxel (PTX) were purchased from Fujifilm Wako Chemicals and Sigma-Aldrich (St. Louis, MO, USA), respectively.

### Spheroid culture

HeLa (1 × 10^4^ cells per well) and HCA-1 (2 × 10^4^ cells per well) cells were seeded in ultra-low attachment (ULA) 96-well U-bottom plates (#MS-9096U; Sumitomo Bakelite, Tokyo, Japan), cultured for 24 h at 37 ℃ in a humidified 5% CO_2_ atmosphere, and formed into a 3D structure. All tests were performed after confirming the formation of single spheroids in each well. Cultures were maintained by replacing 50% of the medium by every two days.

### Cell culture in collagen gel

HeLa cells and spheroids were mixed with 30 mL of cold neutralized collagen type-I gel (Cellmatrix; Nitta Gelatin, Osaka, Japan) containing 1 × minimum essential medium (MEM) and placed on a 6-well plate. After the collagen containing the cells and spheroids had solidified, 2 mL of DMEM with 10% FBS was added to each well.

### Sphere cultures of hCSCs

HeLa cells were suspended as single cells and placed in the ULA 96-well U-bottom plates containing a serum-free DMEM/F12 medium with 20 ng/mL of epidermal growth factor (EGF), 20 ng/ml of basic fibroblast growth factor (bFGF), and 0.4% bovine serum albumin (BSA, Sigma–Aldrich). Cultures were maintained by replacing 50% of the medium by every two days.

### Irradiation

In the microtubes, the cells and spheroids were irradiated with X-rays using an X-ray irradiation device (#MBR-1520R-4; Hitachi Power Solutions, Hitachi, Japan). The X-ray generator was operated at 150 kVp and 20 mA with 0.5 mm Al and 0.3 Cu filters. The dose rate of X-rays was approximately 2.1 Gy/min. Some cells and spheroids in 6-well plates were also irradiated with C-ion scanning beams with an initial energy of 140–200 MeV/n and a spread-out Bragg peak (SOBP) width of 6 cm generated by the ion-beam Radiation Oncology Center in Kanagawa (i-ROCK, Kanagawa Cancer Center, Yokohama, Japan). Some of the irradiations were performed using C-ion passive beams accelerated by the Heavy Ion Medical Accelerator in Chiba (HIMAC) at the National Institutes for Quantum and Radiological Science and Technology (QST, Chiba, Japan). The initial energy of the C-ion beam was 290 MeV/u and the width of the SOBP was 6 cm. The isocenter planes were matched to the center of the SOBP.

### Clonogenic survival assay and crystal violet assay

HeLa cells were irradiated with 0, 2, 4, and 6 Gy of X-rays or 0, 1, 2, and 3 Gy of C-ion beams. Cells were then immediately seeded in flat-bottomed 6-well plates at a predetermined number of cells (X-ray: 200 cells for 0 Gy, 400 cells for 2 Gy, 800 cells for 4 Gy, and 1600 cells for 6 Gy; C-ion beam: 200 cells for 0 Gy, 400 cells for 1 Gy, 800 cells for 2 Gy, and 1600 cells for 3 Gy) and cultured for 10–14 days [16]. Thereafter, cells were washed, fixed with methanol, and stained with 0.5% crystal violet (Kanto Chemical, Tokyo, Japan). After washing and drying the plates overnight, the colonies were counted. The survival curves were fitted to a linear-quadratic (LQ) model: S = exp (− αD − βD^2^) for the X-ray and linear models: S = exp (− aD) for the C-ion beam. S is the survival fraction, and D is the dose expressed in Gray [17]. Statistical analyses were performed using GraphPad Prism 9.3.1 (GraphPad Software, La Jolla, CA, USA).

HCA-1 cells were seeded in 96-well plates at a density of 1 × 10^3^ cells per well and cultured for 10–14 days. Viability was evaluated using a crystal violet assay [18]. Cells were washed, fixed with 4% glutaraldehyde, and stained with 0.5% crystal violet. After washing and drying plates, stained cells were lysed with 100 µL of 1% sodium dodecyl sulfate (SDS), and the absorbance was measured at 570 nm using an iMark microplate reader (Bio-Rad Laboratories, Hercules, CA, USA).

### Cell viability

Cell viability was determined using a Cell Titer-Glo assay (#G9683; Promega, Madison, WI, USA) or a Cell Counting Kit-8 (CCK-8) colorimetric assay (#CK04, Dojindo Molecular Technologies, Kumamoto, Japan). For the Cell Titer-Glo assay, cells were added 25 µL of Cell Titer-Glo 3D were added to the cells in each well, followed by incubation for 30 min at room temperature. Luminescence was measured using a SpectraMax L Plate Reader (Molecular Devices, San Jose, CA, USA). For the CCK-8 assay, a CCK-8 solution was added to each well and the absorbance was measured at 450 nm using an iMark microplate reader after 2 h. Data were normalized to the control wells for each cell culture.

### Immunoblot

Cells were lysed with an ice-cold lysis buffer containing 25 mM of Tris-HCl pH 7.4), 150 mM of NaCl, 1 mM of EDTA, 1% Triton-X, and a protease inhibitor cocktail (Calbiochem, Merck KgaA). Nuclear and cytoplasmic proteins were prepared using a LysoPure™ Nuclear and Cytoplasmic Extraction Kit (Fujifilm Wako Chemicals). Total, nuclear, and cytoplasmic protein concentrations were determined using a Pierce™ BCA Protein Assay Kit (Thermo Fisher Scientific, Waltham, MA, USA) according to the manufacturer’s instructions. Equal amounts of proteins were denatured by heating at 95 ℃ for 5 min with Laemmli sample buffer, separated by SDS-PAGE (Atto, Tokyo, Japan), and transferred to polyvinylidene fluoride membranes (0.45 μm, Millipore, Merck KgaA). After blocking membranes with 5% skim milk, the blots were incubated for 1 h with the following primary antibodies: anti-E-cadherin (E-cad) (#3195; Cell Signaling Technology (CST), Danvers, MA, USA, 1:500), anti-Integrin β1 (ITGB1) (#sc59827; Santa Cruz Biotechnology (SCB), Dallas, TX, USA, 1:500) anti- hypoxia-inducible factor 1α (HIF1α) (#ab51608; Abcam, Cambridge, UK, 1:500), anti-GAPDH (#sc-32233; SCB, 1:500), and anti-TATA-binding protein (TBP; #22006-1-AP; Proteintech, Rosemont, IL, USA, 1:500). Blots were then incubated with goat anti-rabbit or goat anti-mouse secondary antibodies (CST) and visualized with Amersham ECL Prime (Cytiva), followed by chemiluminescence detection using a ChemiDoc Touch imaging system (Bio-Rad). Protein expression levels were evaluated by densitometry using the ImageJ software (https://imagej.nih.gov/ij/).

### Flow cytometric analysis

For cell cycle analysis, cells were harvested, fixed with 70% chilled ethanol, and stained with a FxCycle^TM^PI/RNase Staining Solution (Invitrogen, Thermo Fisher Scientific).

For stem cell analysis, cells were harvested, stained with anti-CD49f (#313602, BioLegend, San Diego, CA, USA) for 30 min on ice, and then stained with Alexa Fluor 488 anti-mouse IgG (#A11029; Invitrogen, Thermo Fisher Scientific) on ice for another 30 min. Cells were also stained with anti-CD44 variant 9 (CD44v9)-PE (#394404, BioLegend) for 30 min on ice.

After washing, measurements were performed using a FACS Canto II cell analyzer (BD Biosciences, San Jose, CA, USA). Data were analyzed using FlowJo software (Treestar, Ashland, OR, USA).

### Sphere-formation assay

For analysis of sphere formation, irradiated HeLa cells were harvested 1 day later and seeded into ULA 96-well U-bottom plates at 100 cells per well. After 3 days of incubation, images of spheres were captured using a Nikon Eclipse Ti inverted microscope with NIS-Elements Advanced Research software (Nikon, Tokyo, Japan), and the viability of spheres was measured using Cell Titer-Glo 3D (Promega).

### Imaging

Images of monolayer cells and spheroids were captured using a Nikon Eclipse Ti inverted microscope (Nikon). For hypoxia imaging, spheroids were stained with the hypoxia probe solution LOX-1 (Medical and Biological Laboratories, Nagoya, Japan), which was quenched by oxygen and increased in response to hypoxic conditions [19, 20]. Images were captured using a fluorescence microscope (#BZ-9000; Keyence, Osaka, Japan).

For immunohistochemistry, cells were fixed and stained using standard protocols [21]. After irradiation, cells were fixed with 4% paraformaldehyde (Fujifilm Wako Chemicals), treated with 0.5% Triton X-100 in PBS, and blocked with 10% normal goat serum (Invitrogen, Thermo Fisher Scientific). The primary antibody used was anti-γH2AX (#05-636; Upstate, Merck KGaA). The secondary antibody used was Alexa Fluor 568 goat anti-mouse IgG (#A11004; Invitrogen, Thermo Fisher Scientific). Cell nuclei were stained with Hoechst 33342 (Fujifilm Wako Chemicals). Images were taken using confocal microscopy (#LSM710; Carl Zeiss Microscopy GmbH, Jena, Germany).

### Statistical analyses

Data are presented as the mean of three minimal independent experiments with corresponding error bars for standard deviation (SD), as indicated in the figure legends. Data analysis was performed by one-way ANOVA followed by the Bonferroni test or Tukey’s test using GraphPad Prism 9.3.1. A *p*-value of less significance was set to indicate statistical significance.

## Results

### Radiosensitivity of cervical adenocarcinoma cell lines in 2D monolayer culture

**Fig. 1:**
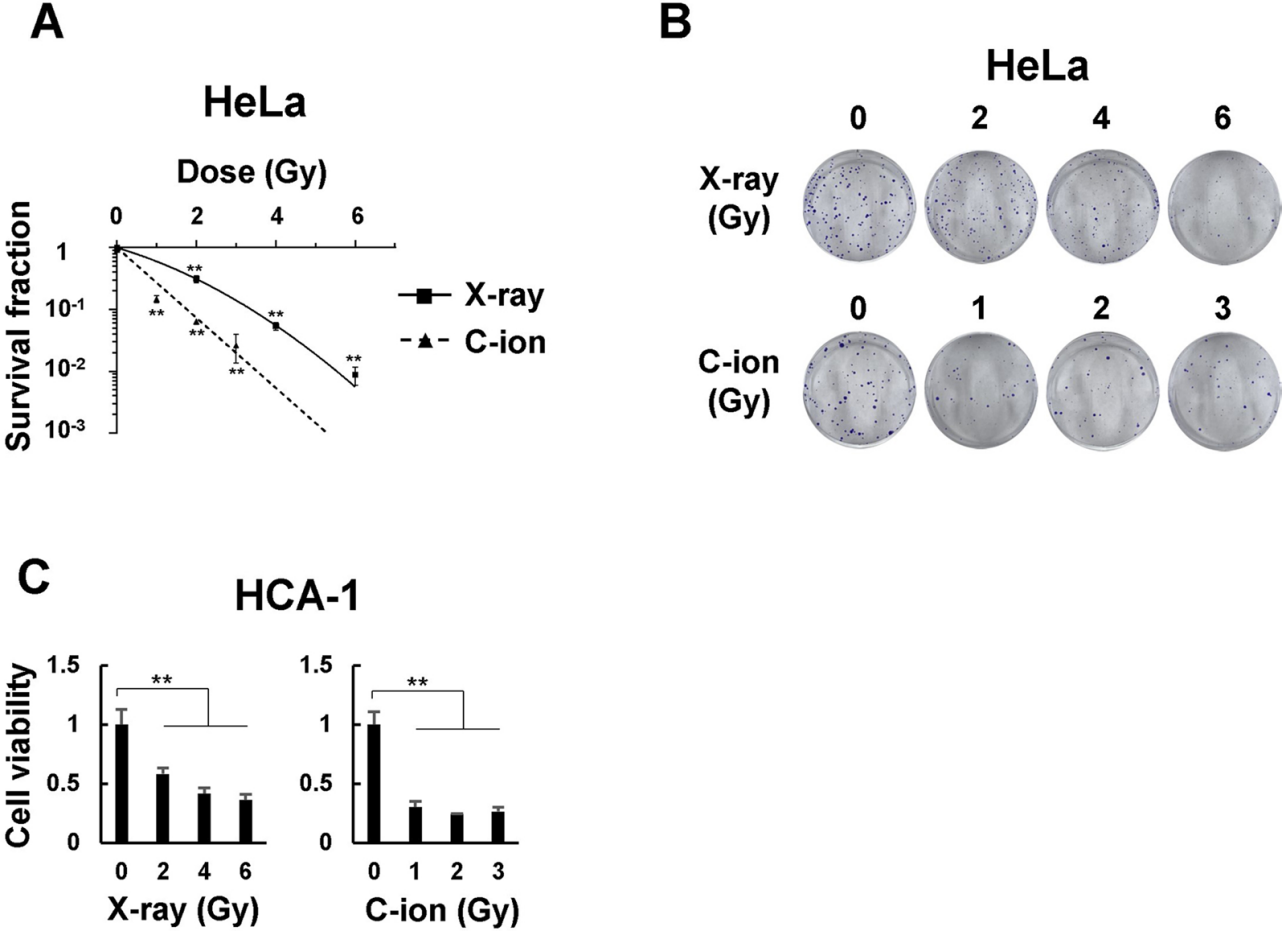
Radiosensitivity of cervical adenocarcinoma cell lines in 2D monolayer culture. **A** HeLa cells irradiated with X-ray or C-ion beams were cultured for 12 days for the clonogenic survival assay. **B** Representative images of HeLa colonies. **C** HCA-1 cells irradiated with X-rays or C-ion beams were cultured for 12 days, and cell viability was determined using a crystal violet assay. The results are presented as the mean ± standard deviation (SD) of three independent experiments. *p < 0.05, **p < 0.01

We first performed a clonogenic survival assay to determine the radiosensitivity of cervical adenocarcinoma cell lines. The surviving fraction of HeLa cells irradiated with X-ray and C-ion beams decreased exponentially in a dose-dependent manner (Fig. 1A). Based on the survival curves in Fig. 1A, the relative biological effectiveness (RBE) value at D_10_ (dose required to kill 90% of the cell) for HeLa cells was approximately 1.92; thus, isoeffective doses that produce the same biological effects by C-ion beam were approximately half-doses of X-rays. Whereas HCA-1 cells were unable to form colonies in this assay (data not shown), and their viability after 10–14 days of culture was evaluated using a crystal violet assay. X-ray and C-ion beams decreased the viability of HCA-1 cells in a dose-dependent manner (Fig. 1B). We could not calculate the RBE at D_10_ for HCA-1 cells because the cell viability of those irradiated with X-ray and C-ion beams declined initially and then began to plateau at the upper 10% survival. Furthermore, we performed a colony formation assay using SiHa cells, a cervical squamous cell carcinoma cell line, and observed a dose-dependent exponential decrease in the viability of SiHa cells irradiated with X-ray and C-ion beams (Additional file 1: Fig. S1). The values of D_10_, D_37_, D_50_, SF_2_ and RBE in HeLa, HCA-1, and SiHa cells are shown in Additional file 7: Table S1.

### Radiosensitivity of cervical adenocarcinoma cells in 2D and 3D spheroid cultures

**Fig. 2:**
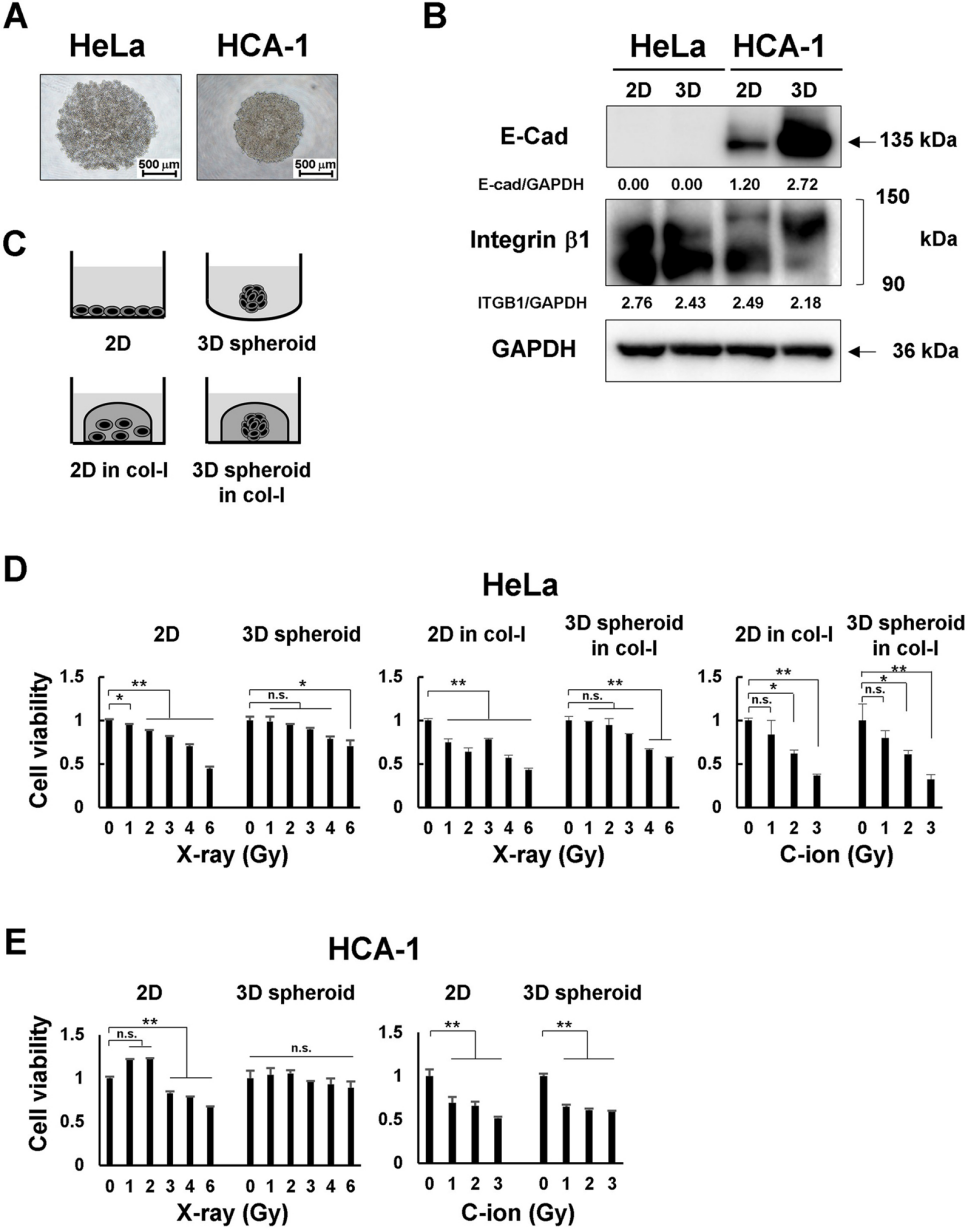
Radiosensitivity of cervical adenocarcinoma cells in 2D and 3D spheroid cultures. **A** Representative images of HeLa and HCA-1 spheroid. HeLa cells formed cell aggregates and HCA-1 cells formed compact spheroids 24 h after seeding. Scale bars: 500 μm. **B** Immunoblot of E-cad and ITGB1. Cancer cells and spheroids were cultured for 48 h after seeding, and total proteins were subjected to SDS-PAGE. GAPDH was used as controls. **C** The illustration to describe 2D or 3D spheroid culture with/without collagen-I. **D**, **E** In 2D cultures, HeLa (**D**) and HCA-1 (**E**) cells were irradiated with X-rays in microtubes, plated onto 96-well plates, and cultured for 4 days. In 3D spheroid culture, HeLa and HCA-1 cells were seeded onto ULA 96-well U-bottom plates and formed spheroids after 24 h. These spheroids were transferred to microtubes, irradiated with X-rays, then returned to 96-well plates and cultured for 4 days. For 2D or 3D spheroid culture in collagen gel, HeLa cells or spheroids were mixed with collagen type-I gel, plate on 35 mm dishes or 6-well plates and cultured at 37℃ for 1 h to solidify the collagen gel. Cells and spheroids were cultured for 4 days after X-ray or C-ion irradiation. Cell viability was measured using Cell Titer-Glo 3D. The results are shown as the mean ± SD of three independent experiments. *p < 0.05, **p < 0.01

To accurately determine the antitumor effects of radiation on cervical adenocarcinoma, we needed to use a model that closely resembles the tumor environment in vivo. Therefore, we used a 3D spheroid models in further experiments. One day after seeding on ULA 96-well U-bottom plate, HeLa and HCA-1 cells formed 3D spheroids (Fig. 2A, Additional file 2: Fig. S2A). The morphology of spheroids can be classified into compact spheroids and cell aggregates depending on the cell type and culture method [22, 23]. In our method, HeLa cells formed cell aggregates and HCA-1 cells formed compact spheroids. We further confirmed the protein levels of E-cad and ITGB1, which are involved in spheroid formation, in HeLa and HCA-1 cells. ITGB1 was expressed in both cell types, whereas E-cad was only expressed in HCA-1 cells. The expression of E-cad was higher in 3D spheroid cultures of HCA-1 cells than in 2D cultures (Fig. 2B). Because HCA-1 cells express not only ITGB1 but also E-cad, they form complete spheroids. Conversely, ITGB1 was expressed in HeLa cells, resulting in initial aggregation. However, E-cad and N-cadherin were not expressed, thus preventing the formation of complete spheroids. Because HeLa spheroids are easily disintegrated when carried to irradiators, these spheroids were embedded in collagen-I gels (Fig. 2C). We next investigated the treatment response to X-ray or C-ion beams in HeLa and HCA-1 cells cultured in 3D spheroids compared with 2D monolayers using the Cell Titer Glo 3D assay kit (Additional file 2: Fig. S2B). Both X-ray and C-ion beams decreased the viability of the two cervical adenocarcinoma cell lines in a dose-dependent manner. As shown in Fig. 2D, HeLa cells tended to show relative resistance to X-rays in 3D spheroid cultures compared with 2D cultures, regardless of collagen embedding. In contrast, C-ion beam significantly decreased the viability of HeLa 2D and 3D spheroid cultures. HCA-1 cells show similar results to those of HeLa cells (Fig. 2E). We also evaluated the effect of collagen embedding. The radiosensitivity of HeLa cells in the collagen-embedded group was higher than that of the unembedded group (Additional file 3: Fig. S3A, B). When 3D spheroids were cultured on collagen, the total number of cells did not change (Additional file 3: Fig. S3C) but were found to infiltrate the collagen gel (Additional file 3: Fig. S3D). This infiltration into the collagen gel may loosen the dense structure of spheroids and increase the radiosensitivity by releasing the hypoxic environment. In addition, our previous studies on collagen culture-induced changes in sensitivities also mention the influence on cell lines in a treatment-dependent manner [24].

### Effects of chemoradiotherapy on cervical adenocarcinoma cells cultured in 2D and 3D systems

**Fig. 3:**
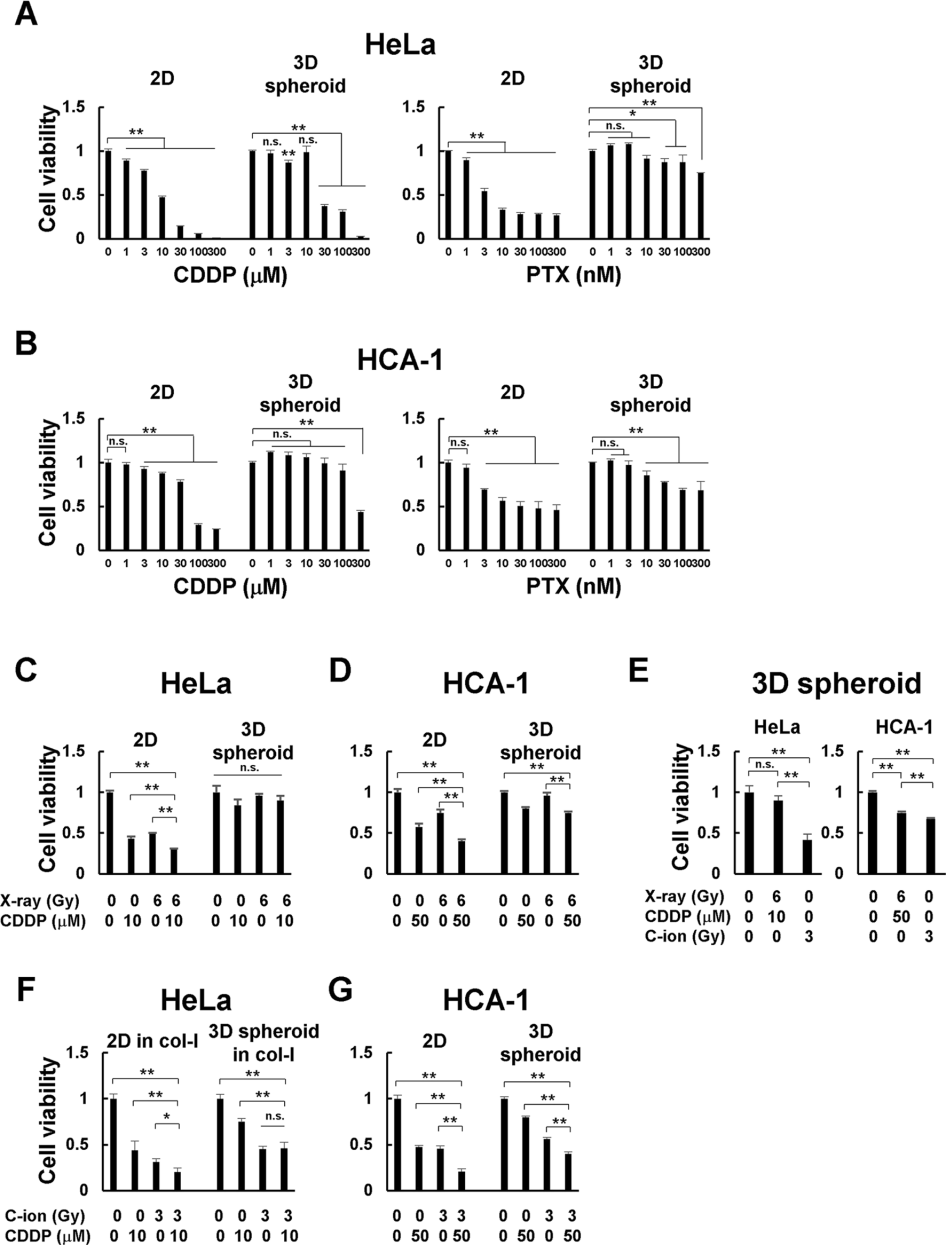
Effects of chemoradiotherapy on cervical adenocarcinoma cells cultured in 2D and 3D systems. **A**, **B** HeLa (**A**) and HCA-1 (**B**) cells were cultured with the indicated doses of cisplatin (CDDP) or paclitaxel (PTX). After 48 h, the media were changed and the cells and spheroids were cultured for another 48 h. **C**, **D** HeLa (**C**) and HCA-1 (**D**) cells were treated with 6 Gy of X-ray and/or CDDP. After 48 h, the media were changed and the cells and spheroids were cultured for another 48 h. (E) Comparison of the effects of CDDP and X-ray in combination with C-ion beam alone in 3D spheroid culture. **F**, **G** HeLa (**F**) and HCA-1 (**G**) cells were treated with 3 Gy of C-ion beam and/or CDDP. After 48 h, the media were changed and cells and spheroids were cultured for another 48 h. Cell viability was measured using Cell Titer-Glo 3D. The results are shown as the mean ± SD of three independent experiments. *p < 0.05, **p < 0.01

To determine whether 2D and 3D culture systems altered drug sensitivity, the antitumor effects of CDDP and PTX, which are used in the clinical treatment of cervical cancer, were compared in 2D and 3D spheroid cultures of HeLa and HCA-1 cells (Additional file 2: Fig. S2C). Both CDDP and PTX drug sensitivities differed between the 2D and 3D culture systems in HeLa (Fig. 3A) and HCA-1 cells (Fig. 3B). The inhibitory concentration 50 (IC50) value against CDDP in 2D-culture HeLa cells in 2D culture was 8.99 µM, while the IC50 value in the equivalent 3D spheroid culture was 23.90 µM, indicating an approximate 2.7-fold higher resistance. The IC50 of PTX in HeLa cells (3.86 nM) was calculated in 2D culture but was almost ineffective in 3D spheroid cultures. IC50 values for CDDP in HCA-1 cells cultured in 2D and 3D spheroid systems were 59.71 nM and 259.26 nM, respectively. HCA-1 cells in 3D spheroid culture were approximately 4.3 times more resistant to CDDP than those in 2D culture. Similar to HeLa cells, the IC50 of PTX in 2D culture could be calculated for HCA-1 cells (35.53 nM), but not for 3D spheroids, because cell viability did not fall below 0%, even when a high dose of PTX was added. Next, we examined the antitumor effects of the combination of CDDP and X-rays on HeLa cells cultured in 2D and 3D spheroids. In both HeLa and HCA-1 cells, the combined effect of X-rays and CDDP was observed in 2D culture, but not in 3D culture (Fig. 3C, D). The antitumor effects of CDDP combined with X-ray or C-ion beams were compared in 3D spheroids. Surprisingly, C-ion beams alone were more effective than the combination of CDDP and X-rays in HeLa and HCA-1 cells (Fig. 3E). Although we found that C-ion beams alone were sufficient in cervical adenocarcinoma cell lines, we hypothesized that the antitumor effect could be further enhanced when CDDP was combined with C-ion irradiation. In 2D cultures, a combined effect was observed in both HeLa and HCA1 cells (Fig. 3F, G). In 3D spheroids, CDDP did not boost the antitumor effect of C-ion beams in HeLa cells (Fig. 3F) but did improve the impact of the carbon beam in HCA-1 cells (Fig. 3G).

### Induction of cell cycle arrest and cell death by X-ray or C-ion irradiation

**Fig. 4:**
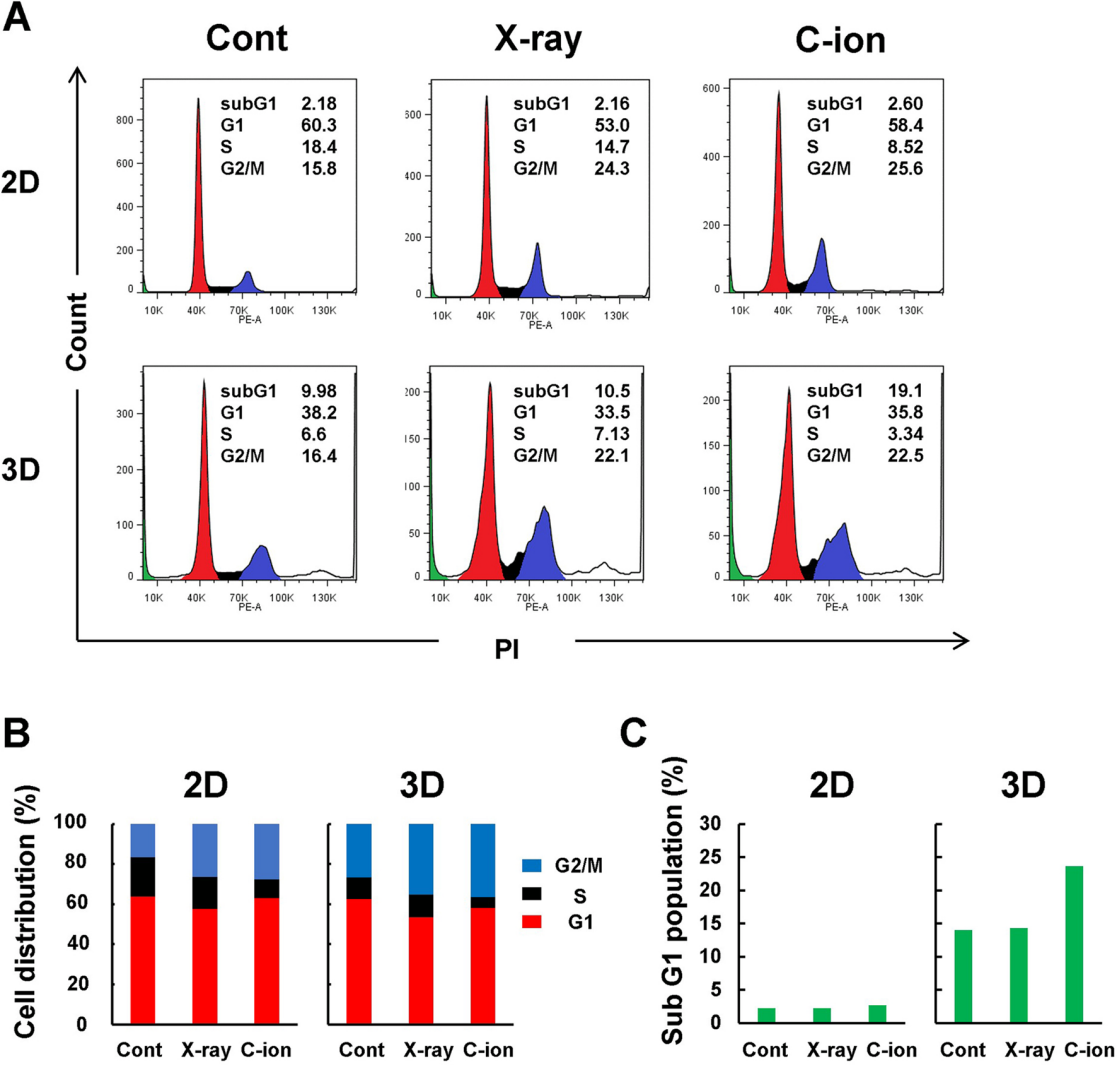
Induction of cell cycle arrests and cell death by the irradiation of X-ray or C-ion beams. **A**, **B** HeLa cells and spheroids were irradiated with 6 Gy of X-ray or 3 Gy of C-ion beams. After 24 h, harvested cells were stained with PI, and flow cytometry was performed. The numbers represent the percentages of each subset. **C** The percentage of HeLa cells in the sub G1 phase

The antitumor effects on cervical adenocarcinoma cells were evaluated by measuring cell viability after exposure to X-rays or C-ion beams. These effects on cell viability may reflect changes in cell death and proliferation. Therefore, we investigated whether cell death and cell cycle arrest were involved in the antitumor effects of X-ray or C-ion irradiation in our culture systems. Cell death and the distribution of irradiated cells collected 24 h after irradiation were assessed by flow cytometric analysis of propidium iodide (PI)-stained cell nuclei. As shown in Fig. 4A, B, X-ray irradiation decreased the percentage of S-phase cancer cells and increased that of G2/M phase cancer cells in HeLa cells. The same trend was observed for the C-ion and X-ray beams, but it was more significant. X-ray and C-ion beams also induced G2/M arrest in 3D spheroids, wherein the percentage decreased compared to that in 2D. The sub G1 phase is representative of cell death, which is evaluated by the percentage of cells in the sub G1 phase. In 2D culture, the ratio of cells in the sub G1 phase was almost unchanged by both X-ray and C-ion beams, which decreased the percentage. However, the number of cells in the sub G1 phase was increased by C-ion irradiation, whereas it remained unchanged by X-ray irradiation (Fig. 4C).

### Radioresistance induced by hypoxia in 3D spheroid cultures

**Fig. 5:**
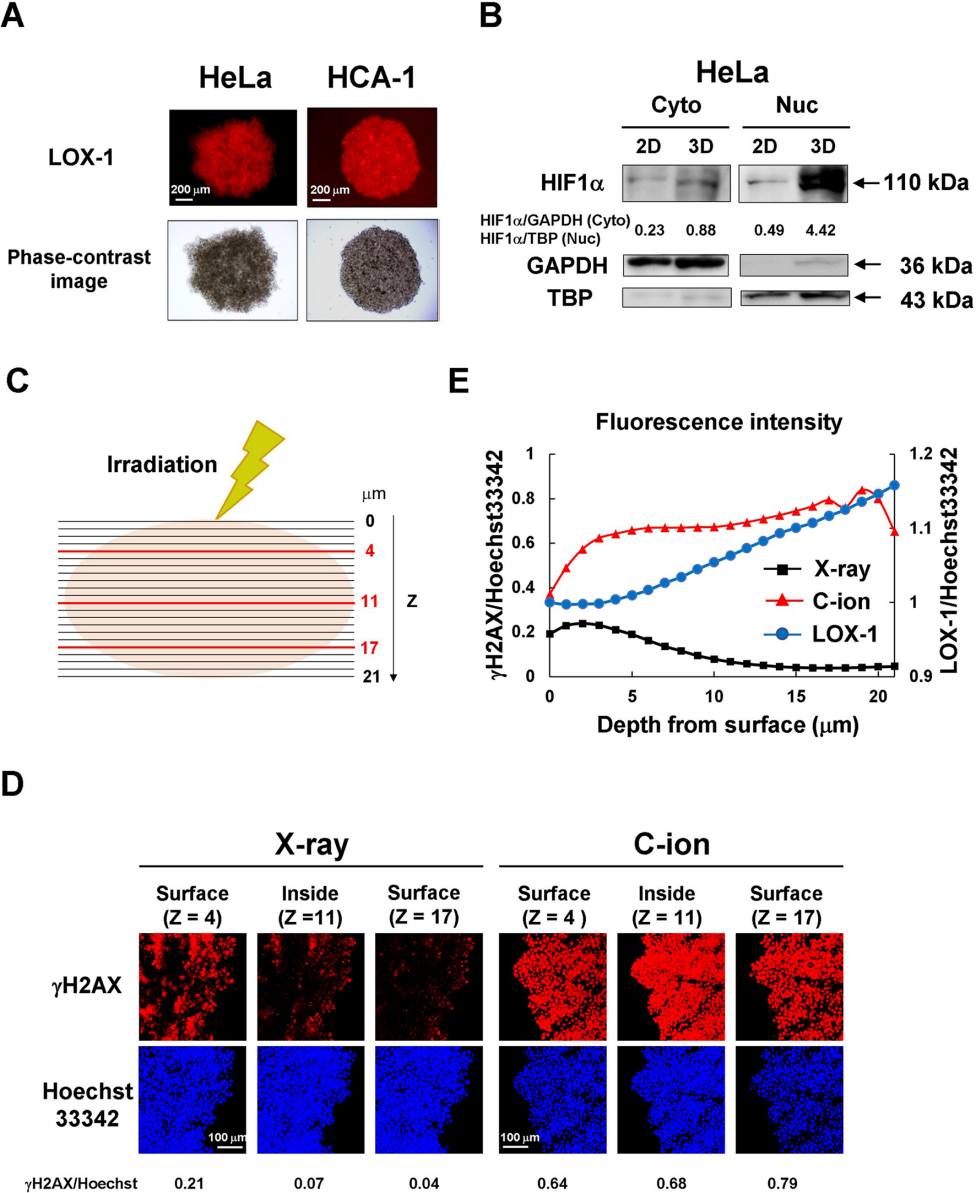
Radioresistance induced by hypoxia in 3D spheroid cultures. **A** Spheroid imaging with LOX-1. Spheroids were cultured for 48 h, stained by the hypoxia probe LOX-1, and then imaged using a fluorescence microscope. Scale bars: 200 μm. **B** Immunoblot of HIF1α. Cancer cells and spheroids were cultured for 48 h after seeding, and the cytoplasmic and nuclear fractions were subjected to SDS-PAGE. GAPDH and TBP were used as controls. **C** The illustration to describe confocal Z-stack images of 3D spheroids. **D** Representative confocal fluorescence microscopy images of γH2AX foci at 30 min after irradiation of HeLa spheroids with 6 Gy of X-rays or 3 Gy of C-ion beams; γH2AX (red) and nuclear DNA stained with Hoechst 33342 (blue). Scale bars: 100 μm. Fluorescence intensities were quantified against DNA content. **E** Fluorescence intensity profiles of γH2AX/Hoechst 33342 and normalized LOX-1/Hoechst 33342 are shown for HeLa spheroids irradiated with 6 Gy of X-rays or 3 Gy of C-ion beams. LOX-1/Hoechst 33342 fluorescence intensities were normalized to the value of Z = 0

Since the antitumor effects of X-rays and anticancer drugs are attenuated in 3D spheroid culture, we hypothesized that hypoxia inside spheroids may have caused resistance. We first evaluated hypoxic conditions in 3D spheroid culture system using the hypoxia probe LOX-1. Cells cultured in a 2D system were not hypoxic because they were negative in LOX-1 staining (Additional file 4: Fig. S4). In contrast, hypoxia inside the spheroid was observed in HeLa and HCA-1 cells when cultured in 3D systems, as shown in Fig. 5A. Next, we confirmed the expression of the hypoxic marker HIF1α in 2D and 3D culture systems. HIF1α was highly expressed in 3D spheroids but barely expressed in 2D cultures. Some HIF-1α was also present in the cytoplasmic fraction but was more abundantly expressed in the nucleus (Fig. 5B). We then visualized DNA double-strand breaks (DSBs) with immunofluorescence staining for phosphorylated H2AX (γH2AX) foci 30 min post-irradiation to examine the effect of radiation in the hypoxic area. Figure 5C shows images of γH2AX immunostaining captured by confocal microscopy. Additionally, the fluorescence intensity of γH2AX against Hoechst 33342 was quantified based on Z-stack images (Additional file 5: Fig. S5) acquired 1 μm from the top to the bottom of the HeLa spheroids, as shown in Fig. 5D. Furthermore, the fluorescence intensity of γH2AX staining was significantly higher with C-ion beams than with the X-ray beams. These results indicated that DNA-DSBs were induced on the spheroid surfaces by X-ray and C-ion irradiation, while they were induced inside the spheroid by the C-ion beams, but not by X-ray beams.

### Involvement of CSCs in radiosensitivity

**Fig. 6:**
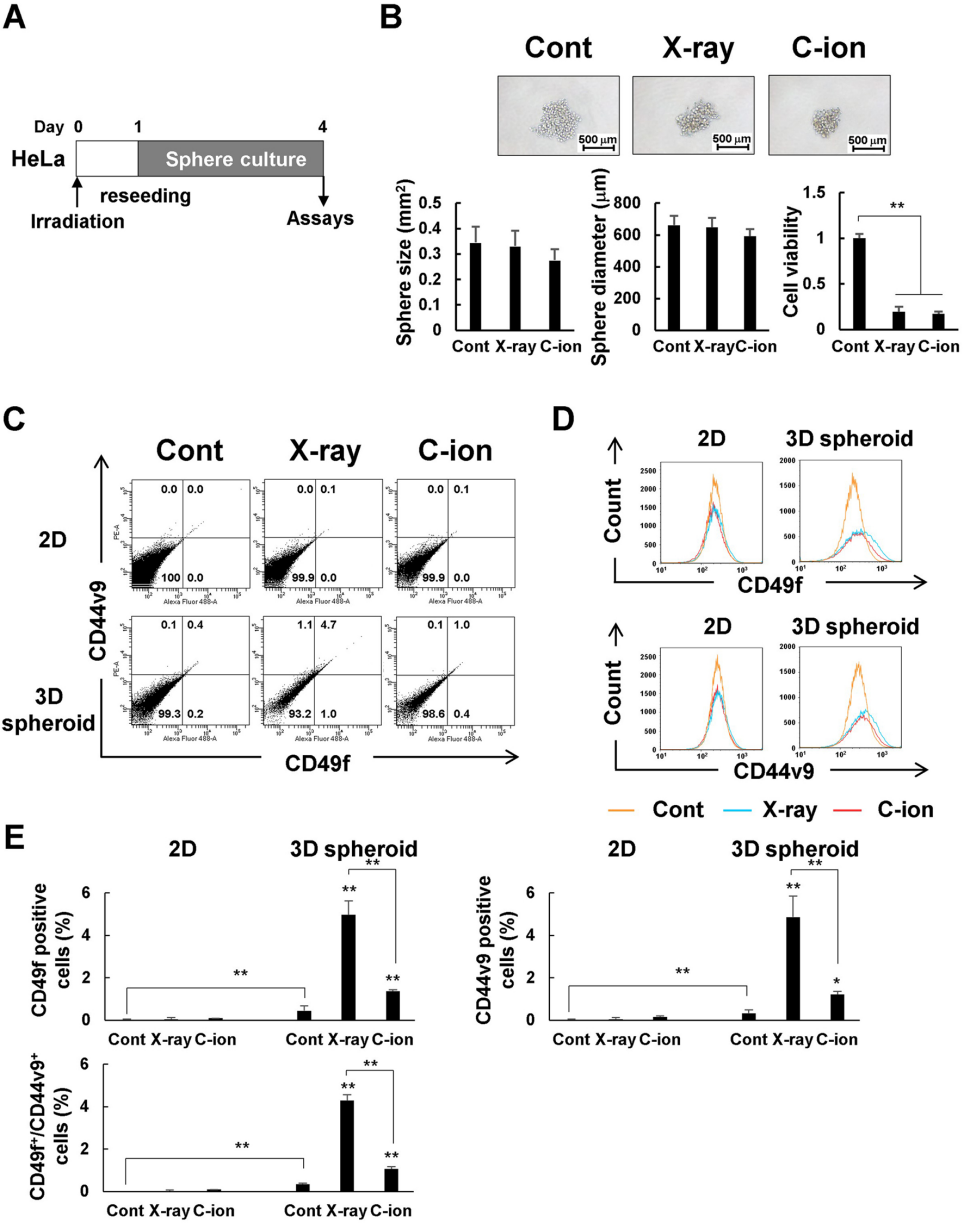
Involvement of CSCs in radiosensitivity. **A** Sphere culture protocols are shown. **B** The spheres during the 3-day incubation period. Sphere images were captured by microscopy. Scale bars: 500 μm. Sphere size and the equivalent circle diameter were calculated with NIS-Elements Advanced Research software. Cell viabilities were measured using Cell Titer-Glo 3D. **C** Cell populations for the CD49f and CD44v9, which are known markers of cervical CSC-like cells after irradiation, were obtained by flow cytometric analysis. **D** Representative FACS histograms showing increased CD49f and CD44v9 in irradiated 3D spheroid compared with 2D systems. **E** Percentages of cells expressing CD49f, CD44v9, or both are shown as a bar graph. Data are presented as the mean ± SD (n = 6), *p < 0.05, ** *p* < 0.01

Finally, we examined the effects of X-ray and C-ion beams on CSCs that exist in hypoxic regions and are resistant to treatment. We used spherical cultures [25] and specific surface markers [26]. As shown in Fig. 6A, X-ray- or C-ion-irradiated HeLa cells were harvested after 1 day and cultured in serum-free spheres for 3 days. Spheres were formed in all cases under X-ray irradiation, C-ion irradiation, and the control (non-irradiated) environment. Cell viability was significantly reduced in spheres formed from the X-ray- and C-ion-irradiated cells (Fig. 6B). We next examined whether HeLa cells cultured in 3D spheroids contained cells expressing the stem cell markers CD49f [26] and CD44v9 [27]. Flow cytometric analysis showed that the percentages of CD49f-positive, CD44v9-positive, and CD49f/CD44v9-positive cells were significantly increased in 3D spheroid culture alone compared to 2D culture (Fig. 6C–E) despite being cultured with serum. Moreover, an apparent increase in CD49f/CD44v9-positive cells was observed in irradiated 3D spheroids compared to non-irradiated ones (Fig. 6C), and this was more significantly in the X-ray-irradiated spheroids (4.7%) than in the C-ion-irradiated spheroids (1.0%). The histogram of CD49f-positive and CD44v9-positive cells shows that these cells did not change after irradiation in 2D systems, but the percentage of stem cell-like cells increased after X-ray and C-ion irradiation in 3D spheroids (Fig. 6D, E). Stem cell-like cells increased after irradiation, but only slightly with C-ion irradiation compared to X-ray irradiation.

## Discussion

Cervical adenocarcinoma has an inferior prognosis because it is resistant to chemoradiotherapy, the primary treatment for locally advanced cases [28]. Therefore, there is an urgent need to develop new treatment methods for overcoming this resistance. In this study, we investigated the efficacy of heavy-particle radiotherapy, which recent clinical trials have suggested is effective against cervical cancer in recent clinical trials. We first examined the effects of X-ray and C-ion irradiation on traditional 2D cultures and found that both treatments decreased cell viability in a dose-dependent manner (Fig. 1). However, previous studies have reported that 2D cultured cancer cell lines overestimate the antitumor effects of treatment [29]. Therefore, it is necessary to use a model that closely resembles the in vivo tumor environment to accurately understand the effects of radiation. In recent years, the advantages of using 3D in vitro models that better mimic in vivo tumor morphology than 2D cultures have been reported [30, 31]. Among 3D culture models, spheroids are the best characterized organotypic models of cancer [32].

Among several methods for generating spheroids, such as suspension culture [33], hanging drops [34], and liquid overlay [32, 35], we used the ULA plate method, which is easy to handle and produces spheroids of uniform size [36]. X-ray irradiation and treatment with anticancer drugs decreased cell viability in a dose-dependent manner in 2D cultures, whereas their effects were significantly attenuated in 3D spheroid cultures (Figs. 2, 3). Moreover, the combination of X-rays and CDDP demonstrated synergistic effects in 2D cultures but not in 3D spheroids. However, C-ion irradiation showed the same antitumor effects in 3D spheroid cultures as in 2D cultures (Fig. 2). These results suggest that 3D cultures may avoid the overestimation of antitumor effects observed in 2D cultures. We used two cervical adenocarcinoma cell lines, HeLa and HCA-1, and observed that HeLa cells were more sensitive to radiation and anticancer drugs than HCA-1 cells (Figs. 2, 3). HeLa cells have wild-type p53, but p53 is inactivated because it is an HPV-18 positive cell. In contrast, HCA-1 has a p53 missense mutation (R273C) according to the Broad institute’s The Cancer Dependency Map portal (https://depmap.org/portal). Although our study’s lack of p53 investigation is a limitation, tumors harboring a p53-R273C gain of function mutations are known to be more aggressive and resistant to therapies [37], suggesting that the difference in cellular sensitivity between HeLa and HCA-1 cells might reflect the status of p53.

Overall, we investigated two possible mechanisms: cell death and the inhibition of cell cycle arrest. Flow cytometric analysis of PI-stained cell nuclei show that 6 Gy of X-rays and 3 Gy of C-ion beams decreased the S-phase fraction and increased the G2/M-phase fraction 24 h after irradiation in 2D culture (Fig. 4A, B), which is consistent with previous reports [38, 39]. The effects of 6 Gy of X-rays and 3 Gy of C-ion beams on cell cycle arrest were almost the same, which is reasonable because the RBE was almost 2. At 24 h post-irradiation, neither X-ray nor C-ion irradiation caused cell death. In contrast, in 3D spheroid cultures, both X-ray and C-ion beams induced G2/M arrest, as in 2D culture, but the effect was slightly attenuated (Fig. 4A, B). As the size of HeLa spheroids exceeded 500 μm in diameter, the sub G1 fraction increased in the control group as the central part of the spheroids became necrotic. Interestingly, the percentage of sub G1 cells was not different in X-ray-irradiated cells relative to the control group, but the number of cells in the sub G1 phase increased in C-ion beam-irradiated cells compared with the control group (Fig. 4C).

Hypoxia inside spheroids has been reported in several studies. It is well known that HIF1α, isolated as a hypoxia-responsive transcriptional factor [40], is not active in normoxia because it is degraded in the proteasome system [41], whereas it is stabilized in hypoxia and becomes active upon nuclear transfer [42]. With LOX-1 staining, our results also confirmed that the interior of the spheroids was hypoxic by LOX-1 staining (Fig. 5A), and HIF1α was more strongly expressed in 3D spheroid cultures than in 2D cultures (Fig. 5B). The expression of HIF1α was higher in the nucleus than in the cytoplasm (Fig. 5B), which is consistent with the fact that HIF1α is stabilized and translocated into the nucleus. Phosphorylation of histone H2AX is one of the earliest changes to occur at the site of DNA-DSB damage and is known to be a specific indicator of the presence of DSBs [43]. However, it has also been used to assess radiosensitivity [44]. We performed the H2AX foci assay to investigate the sensitivity of 3D spheroids to X-rays and carbon beams. Our results indicated that both X-ray and carbon radiation produced DSBs on the surface of the spheroids, whereas DSBs were observed inside the spheroid when exposed to the C-ion beams but not when exposed to X-rays (Fig. 5C, D). The γH2AX foci produced by the C-ion beams were brighter and larger than those produced by X-rays, as previously reported [45]. These results indicate that X-ray and anticancer drug resistance in the inner part of 3D spheroid is due to oxygen effects, whereas heavy-particle radiation is not affected by hypoxia, which is consistent with the results of previous studies on hypoxia in 2D cultures [46, 47].

CSCs present in tumors have been reported to be involved in treatment resistance and metastasis in various cancers [48]. It has also been suggested that X-ray irradiation kills non-CSCs and that surviving more aggressive CSCs can enhance tumor recurrence and metastasis [49, 50]. However, it has been reported that heavy-particle radiation kills CSCs and non-CSCs, resulting in the suppression of cancer invasion and metastasis. We also investigated the role of CSCs in this experimental system. HeLa cells irradiated with X-ray or C-ion beams in 2D systems, harvested 1 day after irradiation, and cultured in spheres for 3 days, formed spheres as well as control (non-irradiated) cells. Cell viability in the spheres was reduced by almost equal amounts by both X-ray and C-ion irradiation (Fig. 6B). These results suggest that the proportion of CSCs among HeLa cells was small. We then examined whether the percentage of stem cells is altered by 3D spheroid culture. FACS analysis revealed a significantly higher enrichment of CSC-like cells expressing CD49f, CD44v9, or both after X-ray irradiation than after C-ion irradiation (Fig. 6C–E). These results suggest that CSC-like cells are resistant to X-rays and selectively kill non-CSCs, resulting in a relative increase in the proportion of CSC-like cells. In contrast, C-ion irradiation kills CSC-like cells and non-CSCs simultaneously, indicating that the change in the proportion of CSC-like cells in the population is relatively small. These results are consistent with previous studies showing that heavy ion radiation kills CSCs that are resistant to X-rays as well as non-CSCs [10, 51]. Furthermore, X-ray irradiation might cause non-CSCs to transform into CSC-like cells, as recently reported [52].

In conclusion, we investigated the antitumor effects of X-ray and C-ion irradiation in 2D and 3D cultures of cervical adenocarcinoma cells. Our findings not only reveal that 3D spheroid culture may be excellent predictive tools for treatment response with more clinically relevant results but also that heavy-particle radiotherapy may be a new therapeutic strategy to overcome the resistance of cervical adenocarcinoma to treatment.

## Supplementary information

**Additional file 1: Figure S1.** Radiosensitivity of cervical squamous cell carcinoma in 2D monolayer cultures. (A) SiHa cells irradiated with X-ray or C-ion beams were cultured for 14 days for the clonogenic survival assay. (B) Representative images of SiHa colonies. (C) SiHa cells were cultured for 4 days after X-ray or C-ion irradiation. Cell viability was measured using a CCK-8 assay kit. The results are shown as the mean ± SD of three independent experiments. *p < 0.05, **p < 0.01. Figure S2. Spheroid formation process and protocols for the treatment of 2D or 3D cultured cervical adenocarcinoma cells with X-ray, C-ion, or anticancer agents. (A) Cell aggregation is triggered by integrin-mediated attachment to ECM molecules, and then the cells aggregate compactly through the involvement of E-cad [23, 53]. (B, C) The schematic diagram to describe the treatment of cervical adenocarcinoma cells cultured in 2D and 3D systems with X-rays, C-ions (B), and anticancer drugs (C). 

**Additional file 2: Figure S2.**Spheroid formation process and protocols for the treatment of 2D or 3D cultured cervical adenocarcinoma cells with X-ray, C-ion, or anticancer agents. (A) Cell aggregation is triggered by integrin-mediated attachment to ECM molecules, and then the cells aggregate compactly through the involvement of E-cad [23, 53]. (B, C) The schematic diagram to describe the treatment of cervical adenocarcinoma cells cultured in 2D and 3D systems with X-rays, C-ions (B), and anticancer drugs (C).

**Additional file 3: Figure S3.**Effect of collagen embedding on radiosensitivity. (A) HeLa cells (5 × 10^3^) were plated onto a 35 mm dishes or embedded in collagen-I gel in a 35 mm dishes, which were followed by X-ray irradiation. After 4 days, cell viability was determined using Cell Titer-Glo 3D. (B) HeLa cells (1 × 10^4^) were seeded onto ULA 96-well U-bottom plates and spheroids were formed after 24 h. Spheroids were transferred to microtubes or embedded in collagen I gel in a 35 mm dishes, which were followed by X-ray irradiation. After 4 days, cell viability was determined using Cell Titer-Glo 3D. (C) After spheroids were formed, they were cultured for another 5 days with or without collagen embedding. Total cell numbers were measured using a Countess Automated Cell Counter. The results are presented as the mean ± SD of three independent experiments. *p < 0.05, **p < 0.01. (D) Images of HeLa spheroids (left: without collagen embedding; right: with collagen embedding) on day 4. Scale bar: 500 μm.

**Additional file 4: Figure S4.**Images of HeLa cells stained with LOX-1 in 2D culture. HeLa cells were cultured in 2D systems for 48 h, stained by LOX-1, and then imaged using a fluorescence microscope. Scale bars: 100 μm.

**Additional file 5: Figure S5.**Confocal fluorescence microscopy Z-stack images of γH2AX foci 30 min after irradiation in HeLa spheroids with 6 Gy of X-rays (A) or 3 Gy of C-ion beams (B). After staining with γH2AX (red), Z-stack images were obtained every 1 μm from the top to the bottom of HeLa spheroids. Scale bar: 100 μm.

**Additional file 6: Figure S6**. The original raw immunoblot results. (A) The original image of Fig. 2B. (B) The original image of Fig. 5B.

**Additional file 7:** **Table S1.** Characteristics of radiation survival curves for HeLa and HCA-1 cells cultured in two-dimensional culture. D_10_: a lethal dose of 10% survival; D_37_: a lethal dose of 37% survival; D_50_: a lethal dose of 50% survival; RBE: relative biological effectiveness; SF_2_: survival fraction after 2 Gy irradiation.

## Data Availability

Data generated by the authors in this study are included in this article and its Additional files.
