# Supplementary figures and images for: Evaluation of X-ray and carbon-ion beam irradiation with chemotherapy for the treatment of cervical adenocarcinoma cells in 2D and 3D cultures
Source: Cancer Cell Int. 2022 Dec 9;22:391. doi: 10.1186/s12935-022-02810-9 (PMC9733259; doi:10.1186/s12935-022-02810-9)

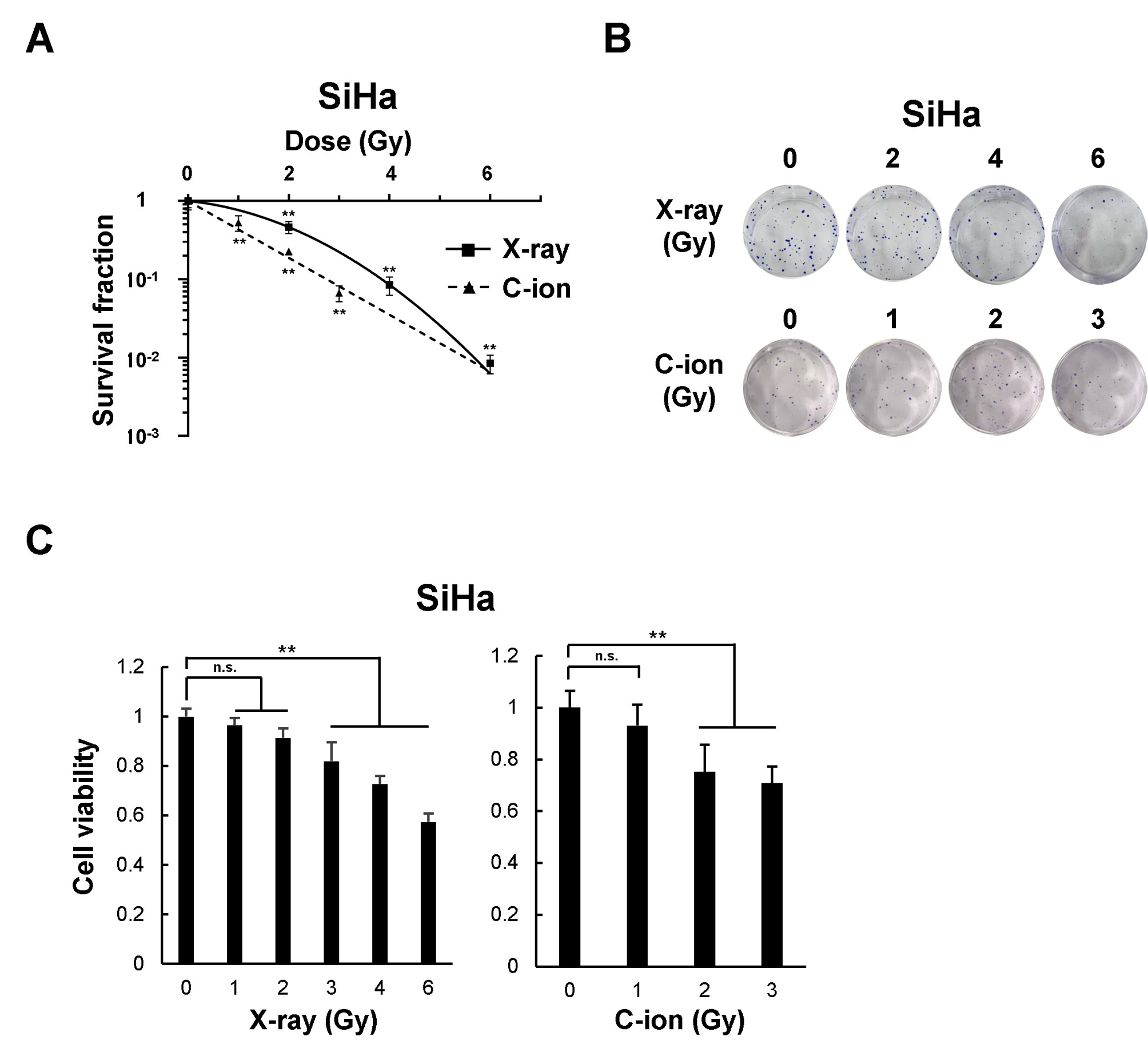

Supplement: Supplementary file 1 — Additional file 1: Figure S1. Radiosensitivity of cervical squamous cell carcinoma in 2D monolayer cultures. (A) SiHa cells irradiated with X-ray or C-ion beams were cultured for 14 days for the clonogenic survival assay. (B) Representative images of SiHa colonies. (C) SiHa cells were cultured for 4 days after X-ray or C-ion irradiation. Cell viability was measured using a CCK-8 assay kit. The results are shown as the mean ± SD of three independent experiments. *p < 0.05, **p < 0.01. Figure S2. Spheroid formation process and protocols for the treatment of 2D or 3D cultured cervical adenocarcinoma cells with X-ray, C-ion, or anticancer agents. (A) Cell aggregation is triggered by integrin-mediated attachment to ECM molecules, and then the cells aggregate compactly through the involvement of E-cad [23, 53]. (B, C) The schematic diagram to describe the treatment of cervical adenocarcinoma cells cultured in 2D and 3D systems with X-rays, C-ions (B), and anticancer drugs (C). [file 12935_2022_2810_MOESM1_ESM.jpg]

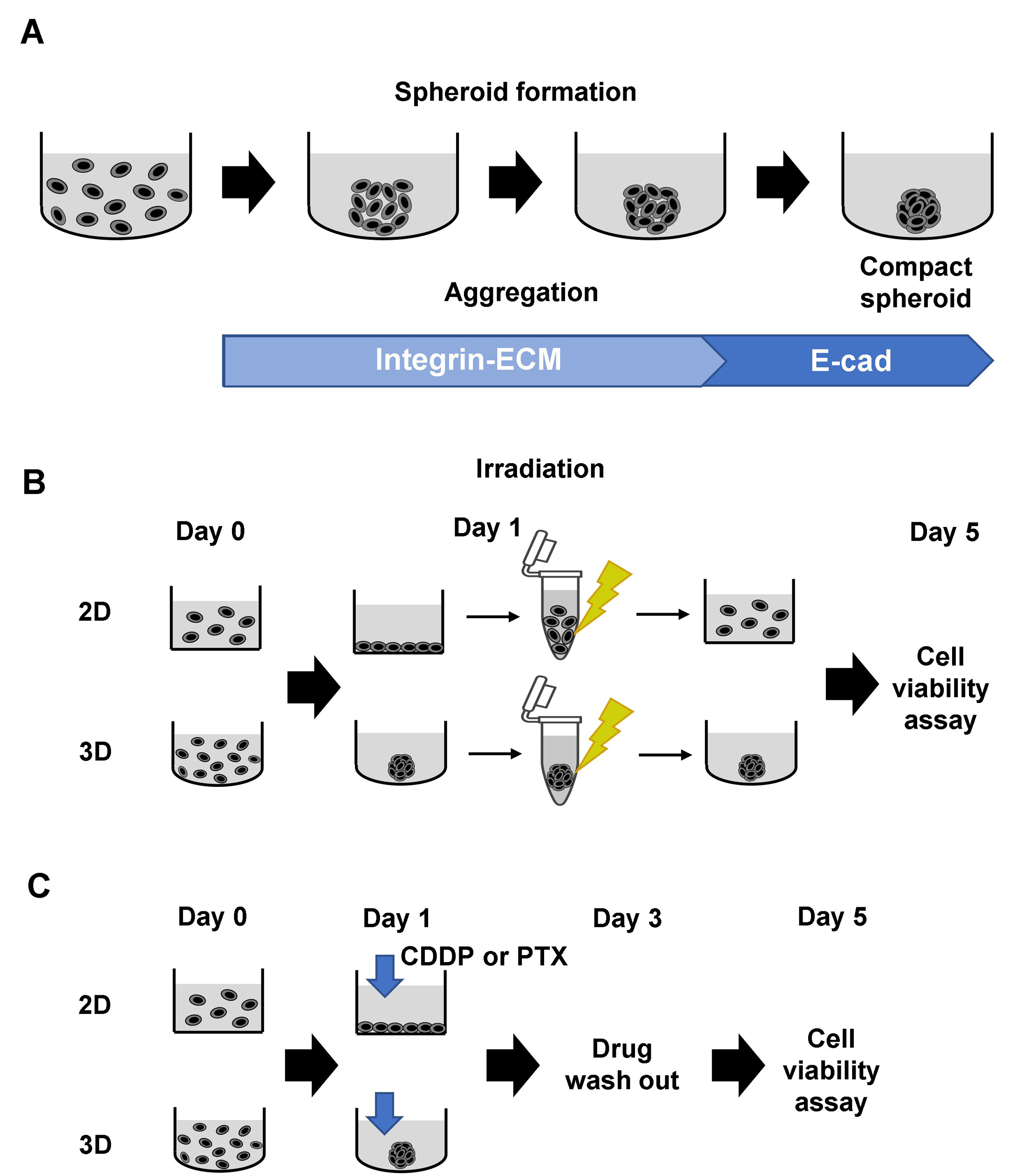

Supplement: Supplementary file 2 — Additional file 2: Figure S2.Spheroid formation process and protocols for the treatment of 2D or 3D cultured cervical adenocarcinoma cells with X-ray, C-ion, or anticancer agents. (A) Cell aggregation is triggered by integrin-mediated attachment to ECM molecules, and then the cells aggregate compactly through the involvement of E-cad [23, 53]. (B, C) The schematic diagram to describe the treatment of cervical adenocarcinoma cells cultured in 2D and 3D systems with X-rays, C-ions (B), and anticancer drugs (C). [file 12935_2022_2810_MOESM2_ESM.jpg]

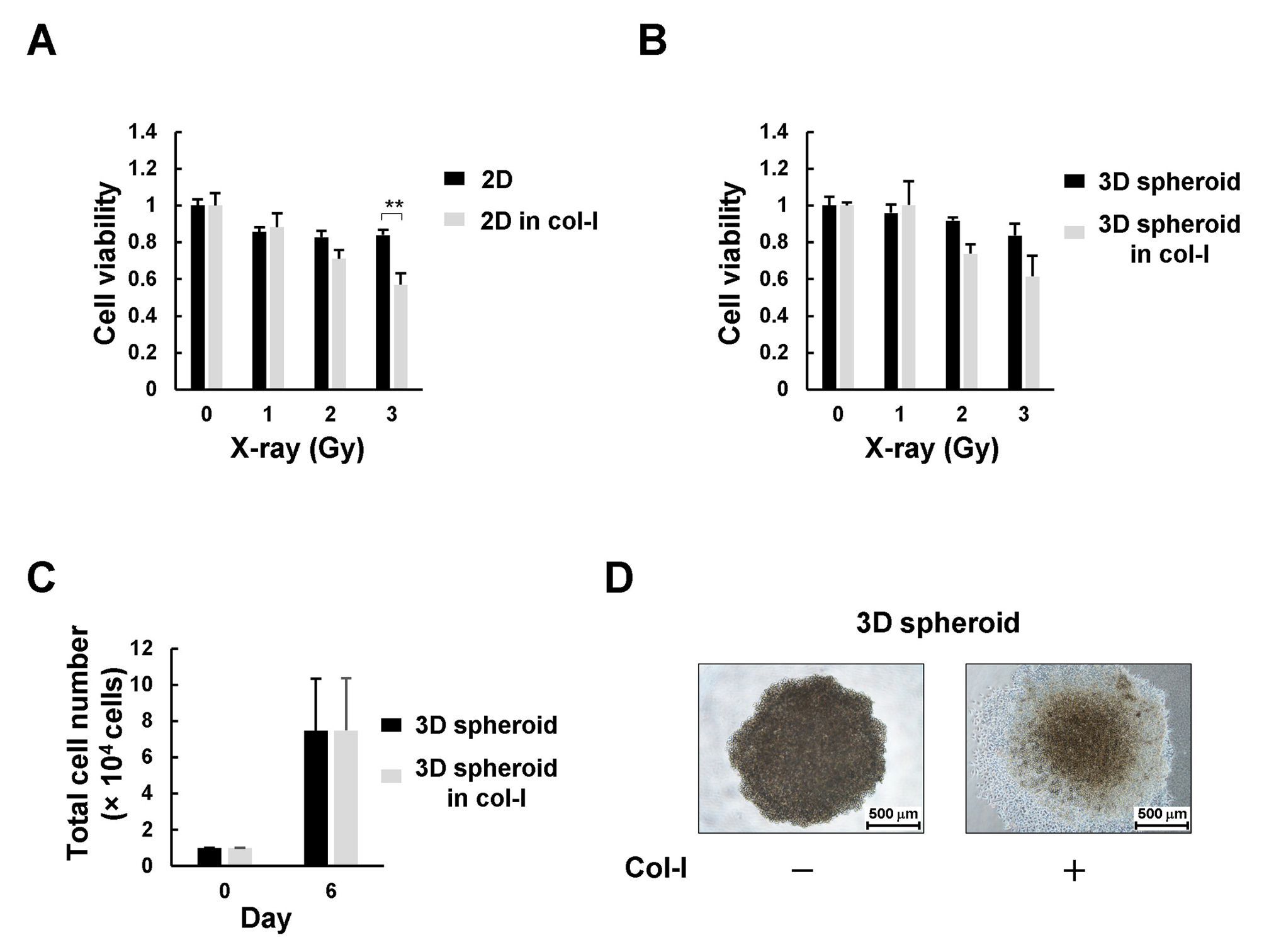

Supplement: Supplementary file 3 — Additional file 3: Figure S3.Effect of collagen embedding on radiosensitivity. (A) HeLa cells (5 × 10^3^) were plated onto a 35 mm dishes or embedded in collagen-I gel in a 35 mm dishes, which were followed by X-ray irradiation. After 4 days, cell viability was determined using Cell Titer-Glo 3D. (B) HeLa cells (1 × 10^4^) were seeded onto ULA 96-well U-bottom plates and spheroids were formed after 24 h. Spheroids were transferred to microtubes or embedded in collagen I gel in a 35 mm dishes, which were followed by X-ray irradiation. After 4 days, cell viability was determined using Cell Titer-Glo 3D. (C) After spheroids were formed, they were cultured for another 5 days with or without collagen embedding. Total cell numbers were measured using a Countess Automated Cell Counter. The results are presented as the mean ± SD of three independent experiments. *p < 0.05, **p < 0.01. (D) Images of HeLa spheroids (left: without collagen embedding; right: with collagen embedding) on day 4. Scale bar: 500 μm. [file 12935_2022_2810_MOESM3_ESM.jpg]

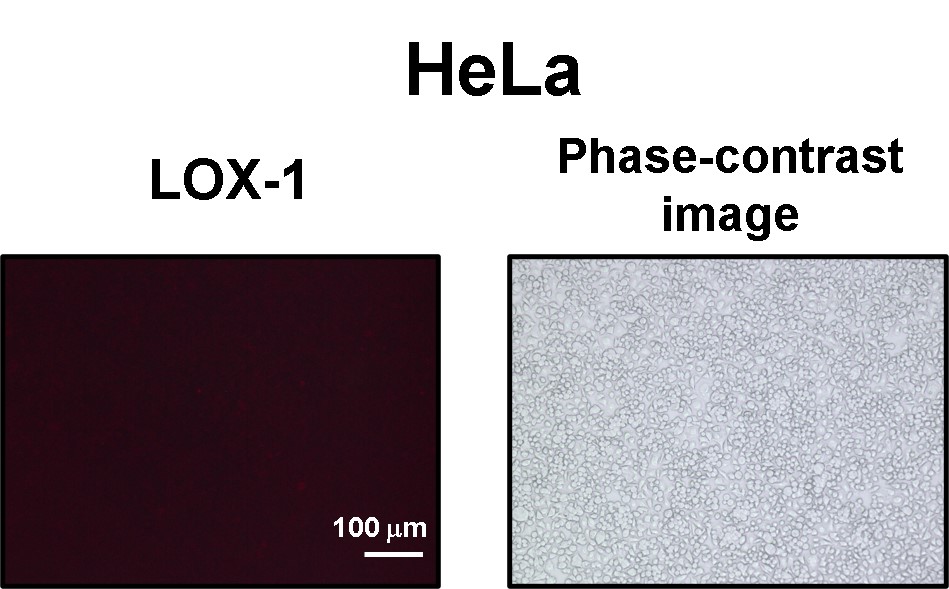

Supplement: Supplementary file 4 — Additional file 4: Figure S4.Images of HeLa cells stained with LOX-1 in 2D culture. HeLa cells were cultured in 2D systems for 48 h, stained by LOX-1, and then imaged using a fluorescence microscope. Scale bars: 100 μm. [file 12935_2022_2810_MOESM4_ESM.jpg]

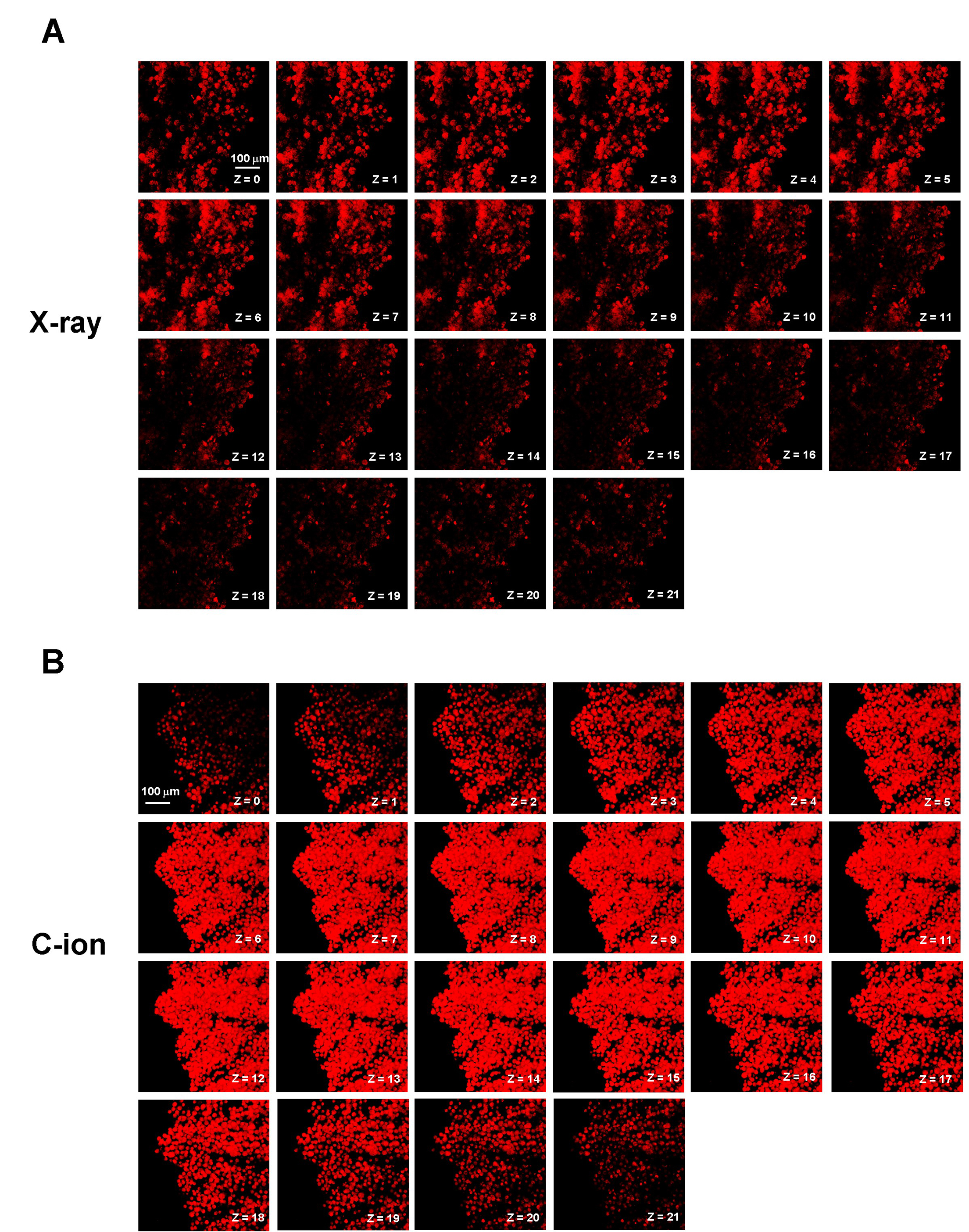

Supplement: Supplementary file 5 — Additional file 5: Figure S5.Confocal fluorescence microscopy Z-stack images of γH2AX foci 30 min after irradiation in HeLa spheroids with 6 Gy of X-rays (A) or 3 Gy of C-ion beams (B). After staining with γH2AX (red), Z-stack images were obtained every 1 μm from the top to the bottom of HeLa spheroids. Scale bar: 100 μm. [file 12935_2022_2810_MOESM5_ESM.jpg]

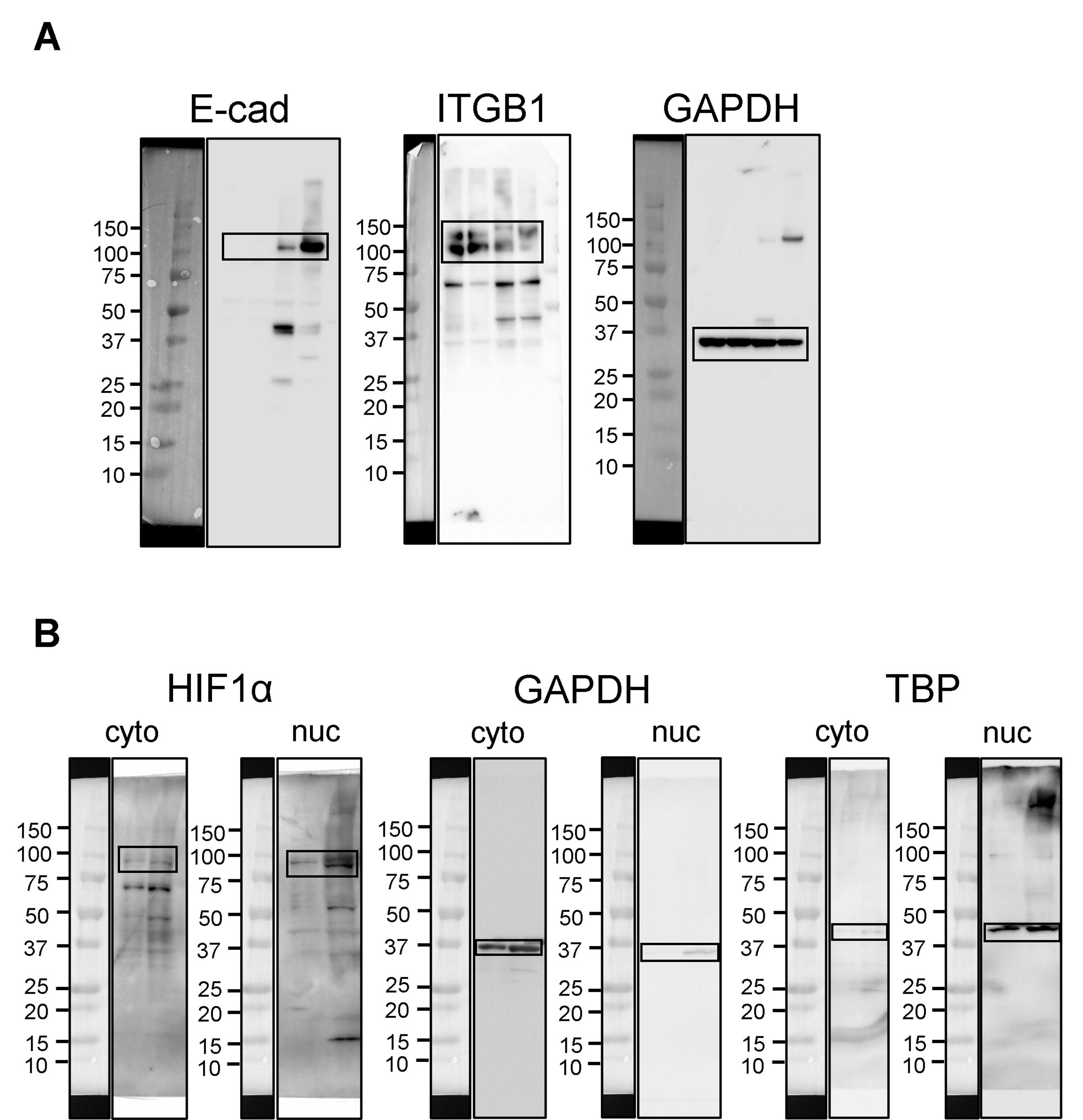

Supplement: Supplementary file 6 — Additional file 6: Figure S6. The original raw immunoblot results. (A) The original image of Fig. 2B. (B) The original image of Fig. 5B. [file 12935_2022_2810_MOESM6_ESM.jpg]
