# Supplementary material for: Evaluation of X-ray and carbon-ion beam irradiation with chemotherapy for the treatment of cervical adenocarcinoma cells in 2D and 3D cultures
Source: Cancer Cell Int. 2022 Dec 9;22:391. doi: 10.1186/s12935-022-02810-9 (PMC9733259; doi:10.1186/s12935-022-02810-9)
Supplement: Supplementary file 7 — Additional file 7: Table S1. Characteristics of radiation survival curves for HeLa and HCA-1 cells cultured in two-dimensional culture. D10: a lethal dose of 10% survival; D37: a lethal dose of 37% survival; D50: a lethal dose of 50% survival; RBE: relative biological effectiveness; SF2: survival fraction after 2 Gy irradiation. [file 12935_2022_2810_MOESM7_ESM.docx]

**Supplemental Table 1.** Characteristics of radiation survival curves for HeLa and HCA-1 cells cultured in two-dimensional culture.

D_10_: a lethal dose of 10 % survival; D_37_: a lethal dose of 37 % survival; D_50_: a lethal dose of 50 % survival; RBE: relative biological effectiveness; SF_2_: survival fraction after 2 Gy irradiation.
